# Supplementary material for: Nutrient Intake with Early Progressive Enteral Feeding and Growth of Very Low-Birth-Weight Newborns
Source: Nutrients. 2022 Mar 11;14(6):1181. doi: 10.3390/nu14061181 (PMC8955398; doi:10.3390/nu14061181)
Supplement: Supplementary file 1 [file nutrients-14-01181-s001.zip › nutrients-1599034-supplementary.pdf]

Table S1. Weekly average nutrient and energy intake in different birth weight groups, median (interquartile range).

Time	Nutrient intake	Birth weight group			*p* value*
		Group 1	Group 2	Group 3	
Week 1**	Total protein, g/kg/day	2.7 (2.3–2.9)	2.2 (1.8–2.4)	1.7 (1.4–2.4)	<0.05[a,b,c]
	Total carbohydrates, g/kg/day	9.3 (8.1–10.3)	7.7 (6.3–9.0)	6.5 (5.2–8.8)	<0.01[a,b]
	Total fat, g/kg/day	2.2 (1.8–3.0)	2.1 (1.5–2.6)	1.8 (1.3–2.8)	0.451
	Total kilocalories, kg/day	65 (56–79)	58 (46–73)	48 (37–68)	0.002[b]
Week 2	Total protein, g/kg/day	3.2 (2.6–3.5)	3.5 (3.0–3.9)	3.7 (3.3–3.9)	<0.05[a,b]
	Total carbohydrates, g/kg/day	12.6 (11.4–14.1)	13.8 (12.7–15.2)	14.2 (12.4–15.1)	<0.05[a,b]
	Total fat, g/kg/day	5.4 (4.8–6.2)	5.7 (5.1–6.6)	5.4 (4.6–6.6)	0.683
	Total kilocalories, kg/day	118 (104–128)	125 (116–134)	128 (106–142)	0.145
Week 3	Total protein, g/kg/day	3.7 (3.0–4.0)	3.7 (2.8–4.0)	3.9 (3.3–4.1)	0.172
	Total carbohydrates, g/kg/day	15.2 (13.3–16.2)	15.9 (13.8–16.5)	16.1 (14.7–16.7)	0.084
	Total fat, g/kg/day	6.1 (5.4–7.4)	6.6 (5.8–7.6)	6.5 (5.8–7.6)	0.398
	Total kilocalories, kg/day	133 (118–144)	135 (127–147)	142 (129–150)	0.123
Week 4	Total protein, g/kg/day	3.5 (3.1–3.8)	3.3 (2.6–3.8)	3.5 (3.1–3.9)	0.709
	Total carbohydrates, g/kg/day	15.8 (13.2–16.6)	16.0 (13.6–16.6)	15.9 (15.0–16.9)	0.512
	Total fat, g/kg/day	6.1 (5.2–7.6)	6.3 (5.7–7.1)	5.8 (5.4–6.6)	0.489
	Total kilocalories, kg/day	137 (119–150)	134 (123–147)	135 (128–142)	0.981

* a – Group 1 vs Group 2, b – Group 1 vs Group 3, c – Group 2 vs Group 3; ** Week 1 intake is not accurate due to lack of mother's milk composition data.

Table S2. Weekly average nutrient and energy intakes in appropriate for gestational age (AGA) and small for gestational age SGA subgroups, median (interquartile range)

Time	Nutrient intake	Subgroup		*p* value
		**AGA (*n*=108)**	**SGA (*n*=12)**	
Week 1**	Total protein, g/kg/day	2.3 (1.8–2.7)	1.8 (1.3–2.7)	0.108
	Total carbohydrates, g/kg/day	8.2 (6.3–9.6)	7.6 (4.3–9.7)	0.554
	Total fat, g/kg/day	2.2 (1.6–2.9)	1.4 (0.9–2.7)	0.071
	Total kilocalories, kg/day	60 (47–74)	51(28–73)	0.166
Week 2	Total protein, g/kg/day	3.4 (2.9–3.8)	3.7 (2.7–4.1)	0.783
	Total carbohydrates, g/kg/day	13.6 (12.0–14.9)	13.5 (12.7–16.1)	0.515
	Total fat, g/kg/day	5.7 (4.8–6.4)	5.5 (4.4–6.2)	0.578
	Total kilocalories, kg/day	123 (108–134)	126(102–138)	0.917
Week 3	Total protein, g/kg/day	3.7 (3.2–4.0)	3.7 (2.6–4.0)	0.611
	Total carbohydrates, g/kg/day	15.8(14.3–16.6)	14.7 (13.9–16.2)	0.258
	Total fat, g/kg/day	6.3 (5.6–7.4)	6.2 (5.3–8)	0.969
	Total kilocalories, kg/day	135 (127–146)	142 (115–153)	0.813
Week 4	Total protein, g/kg/day	3.4 (3.1–3.8)	3.6 (2.0–4.0)	0.907
	Total carbohydrates, g/kg/day	16.0 (14.3–16.6)	15.2 (10.1–16.6)	0.206
	Total fat, g/kg/day	6.1 (5.4–7.1)	6.5 (5.1–7.4)	0.898
	Total kilocalories, kg/day	136 (125–146)	136 (97–155)	0.815

** Week 1 intake is not accurate due to lack of mother's milk composition data.
